# Mitochondrial dysfunction and applications of mitochondrial-targeted delivery systems in atherosclerosis

**DOI:** 10.1080/10717544.2026.2627689

**Published:** 2026-02-10

**Authors:** Yanfang Liu, Nan Luo, Xin Xi, Jinxia Hou, Xiaolu Li, Mingdeng Xia, Tao Yu, Yanyan Yang, Yong Liu

**Affiliations:** a Department of Ultrasound, Qingdao Hiser Hospital Affiliated of Qingdao University, Qingdao, Shandong, People’s Republic of China; b Department of Immunology, School of Basic Medicine, Qingdao University, Qingdao, Shandong, People’s Republic of China; c Dental Department, Qingdao Traditional Chinese Medicine Hospital, Qingdao Hiser Hospital Affiliated of Qingdao University, Qingdao, Shandong, People’s Republic of China; d Institute for Translational Medicine, The Affiliated Hospital of Qingdao University, Qingdao, Shandong, People’s Republic of China

**Keywords:** Atherosclerosis, mitochondria, therapeutic agents, targeted drug delivery, Nanomedicine

## Abstract

Atherosclerosis, a chronic inflammatory disease, is pathologically associated with mitochondrial dysfunction. Mitochondria contribute to oxidative stress, vascular endothelial dysfunction, and chronic inflammatory cascades through pathways such as dynamic imbalance, abnormal epigenetic regulation, disruption of multi-organelle communication, and dysregulation of cell death signaling. Targeting mitochondria has therefore emerged as a promising therapeutic strategy beyond conventional treatments , which often fail to address this underlying pathology. Recent advances in nanomaterials enable precise mitochondrial intervention. Although conventional therapies such as statins and anti-inflammatory drugs can partially mitigate symptoms, they do not directly correct mitochondrial abnormalities and are often limited by systemic side effects. Recent progress in nanotechnology has enabled the development of mitochondria-targeted delivery systems, including liposomes, polymeric nanoparticles, and biomimetic carriers. These platforms enhance mitochondrial accumulation by incorporating targeting motifs or exploiting the negative mitochondrial membrane potential and specific interactions with outer membrane proteins. Among these, TPP⁺-modified liposomes can target the mitochondrial matrix via electrostatic interactions, effectively delivering drugs such as coenzyme Q10 to mitochondria, offering notable clinical potential. Moreover, Szeto-Schiller 31, which targets mitochondrial electron transport chain repair and reduces the secretion of inflammatory cytokines, has entered Phase II clinical trials. This review discusses the mechanistic role of mitochondrial dysfunction in atherosclerosis and evaluates the application of mitochondria-targeted delivery systems in atherosclerosis therapy. It also highlights the challenges these systems face, including issues related to delivery efficiency, biosafety, and targeting specificity. By linking molecular mechanisms with translational innovation, it highlights the significant potential of mitochondrial-targeted therapies.

## Graphical abstract

**Figure:**
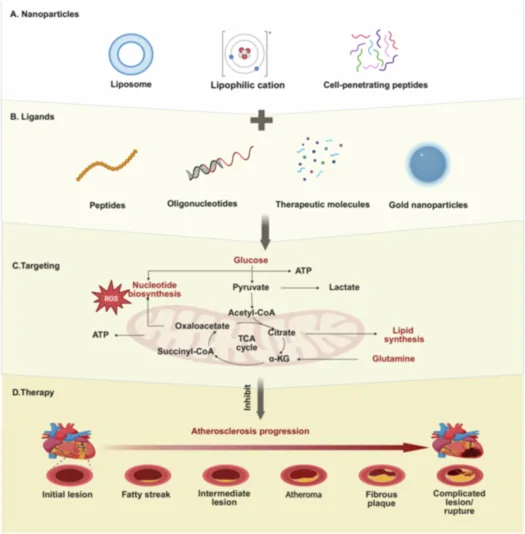

**Mitochondrial-targeted delivery system strategy.** Atherosclerosis is pathologically linked to mitochondrial dysfunction. Targeted nanomaterials have made significant breakthroughs in mitochondrial intervention through innovative delivery strategies, such as liposomes, polymeric nanoparticles, and biomimetic membrane carriers. These systems achieve precise delivery by integrating mitochondrial homing motifs and utilizing either the negative potential of the mitochondrial membrane or the specific binding of outer membrane proteins.

## 1 Introduction

Cardiovascular diseases (CVDs) have remained the leading cause of global mortality over the past decade, imposing substantial health and economic burdens (Wu et al. 2022). Atherosclerosis (AS), a multifactorial chronic inflammatory disease characterised by the deposition of lipid-laden cellular debris and the formation of atherosclerotic plaques in arterial walls, is the primary driver of CVDs (Cheng et al. 2023; Tasouli-Drakou et al. 2025). The pathogenesis of AS involves dysregulated lipid metabolism, oxidative stress, immune dysfunction, and impaired tissue repair responses to injury, with its incidence increasing alongside an aging population (Falk 2006; Libby et al. 2019). Current therapeutic strategies, such as statins, antiplatelet agents, and vascular interventions, have demonstrated limited efficacy in reversing the complex pathophysiological mechanisms of AS or curing the disease. These treatments primarily focus on symptom management and risk factor modification (Orekhov et al. 2020). To date, no pharmacological therapy has demonstrated curative efficacy for AS (Orekhov et al. 2020). As a result, research into the regulatory mechanisms and key therapeutic targets of AS remains a major focus for clinical management.

Mitochondrial dysfunction plays a pivotal role in AS progression by altering cellular metabolism and respiration, leading to excessive reactive oxygen species (ROS) production and oxidative stress (Orekhov et al. 2020; Salnikova et al. 2021). Impaired mitochondrial activity exacerbates endothelial dysfunction, promotes inflammation, and accelerates plaque vulnerability (Buchke et al. 2022). In endothelial cells, mitochondrial dysfunction exacerbates oxidative stress, activates mitophagy pathways, disrupts nitric oxide metabolism and cytosolic calcium cycling, induces inflammatory responses, accelerates senescence, and promotes endothelial cell apoptosis or necrosis. In vascular smooth muscle cells (VSMCs), mitochondrial dysfunction may stimulate the transition of VSMCs from a contractile phenotype to a synthetic phenotype, leading to proliferation and migration to injured sites. In macrophages, genetic variations in mitochondrial DNA (mtDNA) and defective mitophagy may result in altered innate immune responses and subsequent development of a sustained inflammatory state (Shemiakova et al. 2020). Emerging evidence highlights mitochondrial abnormalities as key contributors to AS pathogenesis, underscoring their potential as therapeutic targets (Salnikova et al. 2021).

**Figure 1.**
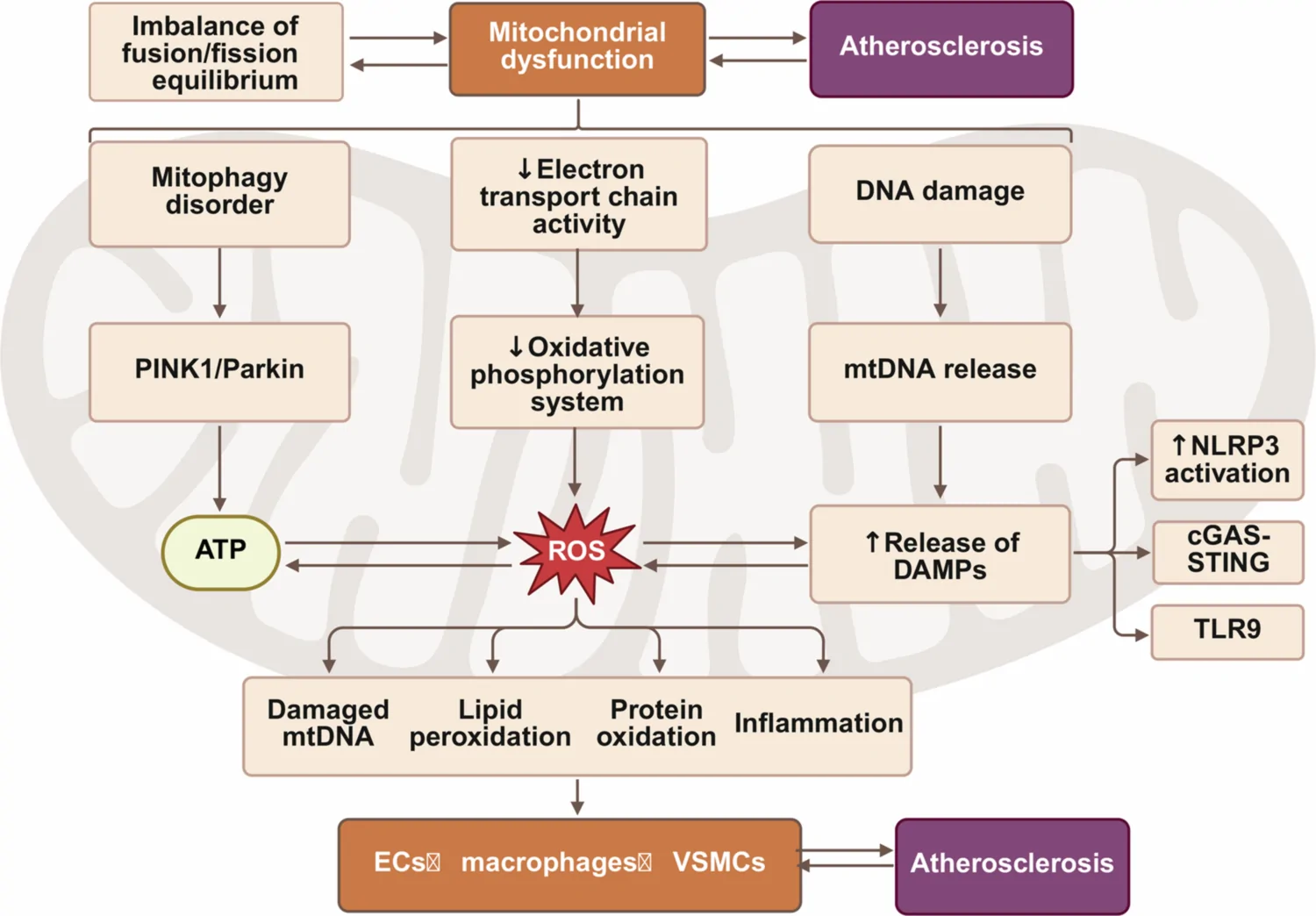
Mitochondrial dysfunction and atherosclerosis. M Mitochondrial dysfunction serves as a central driver in the initiation and progression of atherosclerosis. This process is mediated by three key mechanisms: excessive generation of mtROS, mitochondrial DNA damage, and dysregulation of mitophagy. These disturbances contribute to a self-perpetuating pathological cycle, resulting in vascular endothelial injury, chronic inflammation, and plaque instability, which may lead to rupture.

As research advances, the mechanisms by which mitochondria contribute to cardiovascular pathology are becoming increasingly clear. Numerous potential therapeutic drugs for A including statins and anti-inflammatory agents, are currently being explored. However, traditional drugs lack specificity for mitochondrial targeting and may affect other cellular compartments. Therefore, a targeted strategy is essential for precisely repairing mitochondrial function. Mitochondrial-targeted drug delivery represents one of the most promising therapeutic strategies for atherosclerosis. Ongoing development of mitochondrial-targeting therapies is critical to ensuring long-term efficacy in atherosclerosis treatment (Shemiakova et al. 2020). Consequently, gene therapy and nanotechnology are regarded as promising novel approaches (Shemiakova et al. 2020). Gene therapy leverages cutting-edge technologies, such as CRISPR-Cas9 gene editing, mitochondrial transfer, and RNA interference, to directly repair or replace damaged mtDNA (Wang et al. 2025). This approach offers a novel and promising strategy for the treatment of atherosclerosis. Nanomaterials offer innovative solutions for mitochondrial-specific drug delivery, with lipid-soluble cationic systems, liposomes, and peptide-based nanocarriers demonstrating significant potential in transporting therapeutic payloads to mitochondrial targets (Buchke et al. 2022). These advanced delivery systems enable precise mitochondrial intervention, addressing the organelle-specific pathological alterations that drive atherosclerotic progression. This approach holds transformative potential for developing next-generation therapies targeting mitochondrial dysfunction in cardiovascular diseases. The central role of mitochondrial dysfunction in AS pathogenesis is summarised in Figure 1.

## 2 Mitochondrial dysfunction and atherosclerosis

### 2.1 Mitochondrial structure

Mitochondria consist of four functional units: the outer membrane, the intermembrane space, the inner membrane, and the matrix (Alevriadou et al. 2017; Protasoni and Zeviani 2021). The inner membrane is rich in membrane proteins and generates a proton gradient through the electron transport chain, which dominates adenosine triphosphate (ATP) synthesis (Scheffler 2001; Yu et al. 2025). Mitochondria form a dynamic and interconnected network whose structural organisation directly influences functional capacity (Westermann 2002). This complex network is regulated by intricate crosstalk between mitochondrial fusion and fission processes, which collectively maintain cellular homoeostasis (Parra et al. 2011). Mitochondria are the primary bioenergetic hubs of the cell, coupling two core processes of energy transduction. The tricarboxylic acid (TCA) cycle generates reducing equivalents (NADH/FADH₂) and a small amount of ATP (Du et al. 2025). These electron carriers fuel the electron transport chain (ETC) to establish a proton-motive force across the inner mitochondrial membrane, which ATP synthase uses to produce the majority of cellular ATP via chemiosmotic phosphorylation (Vercellino and Sazanov 2022; Du et al. 2025). As the terminal site of oxidative catabolism, mitochondria integrate pathways such as fatty acid *β*-oxidation to generate acetyl-CoA for the TCA cycle (Vercellino and Sazanov 2022). Additionally, mitochondria support anabolism by supplying TCA cycle intermediates as biosynthetic precursors for amino acids, nucleotides, and lipids. Mitochondria also play a significant role in atherosclerosis by regulating apoptosis, calcium signalling, and inflammation (Szewczyk et al. 2015; Alevriadou et al. 2017; Liu et al. 2025).

### 2.2 Mitochondrial dysfunction and atherosclerosis

Accumulating evidence highlights the critical role of mitochondrial dysfunction in the initiation and progression of atherosclerosis (Peng et al. 2019). The hallmark of mitochondrial dysfunction involves a triad of bioenergetic, antioxidant, and regulatory disturbances, characterised by reduced ATP synthesis, dysregulated cell death pathways, and increased reactive oxygen species (ROS) production (Zinovkin and Zamyatnin 2019).

#### 2.2.1 Mitochondrial ROS in atherosclerosis

Mitochondrial dysregulation contributes to atherosclerosis by altering cellular metabolism and respiration, accompanied by excessive mitochondrial ROS (mtROS) generation, which drives oxidative stress (Tracy et al. 2021; Tokuyama and Yanagi 2023). These processes are intrinsically linked to vascular injury and chronic inflammation in atherosclerotic plaques (Salnikova et al. 2021). Oxidative phosphorylation (OXPHOS), the primary ATP-producing pathway in mitochondria, inherently generates mtROS as a byproduct of electron leakage during respiratory chain activity (Liu et al. 2025). However, dysfunctional mitochondria exacerbate mtROS accumulation, creating a self-perpetuating cycle of oxidative damage that disrupts cellular redox homoeostasis (Zorov et al. 2006; Zhou et al. 2022). A critical pathological cascade arises from mitochondrial membrane potential (ΔΨm) instability and redox imbalance, which trigger the ROS-induced ROS release mechanism (Zorov et al. 2000; Zorov et al. 2014). When mtROS exceed a critical threshold, opening of the mitochondrial permeability transition pore (mPTP) or inner membrane anion channel collapses ΔΨm, hyperactivating the electron transport chain and amplifying ROS production (Zorov et al. 2000; Robichaux et al. 2023). Cytosolic ROS then acts as a secondary messenger, activating adjacent mitochondria via ROS-induced ROS release (RIRR) and establishing a feedforward loop that accelerates mitochondrial dysfunction and endothelial injury (Zorov et al. 2014; Zinovkin and Zamyatnin 2019). The primary mechanism involves the rapid, diffusion-controlled reaction between nitric oxide (NO) and superoxide anion (O₂⁻) to form peroxynitrite (ONOO⁻), a potent oxidising and nitrating agent capable of modifying LDL, even in the presence of endogenous antioxidants (Li et al. 2014). ONOO⁻ mediates lipid peroxidation within LDL particles, generating reactive aldehydes that adduct to apolipoprotein B-100, and promotes nitration of protein tyrosine residues, forming 3-nitrotyrosine, a specific marker of reactive nitrogen species (RNS) activity in atherosclerotic lesions (Zmijewski et al. 2005; Li et al. 2014). This modified LDL is recognised by scavenger receptors such as LOX-1, leading to uncontrolled uptake by macrophages and foam cell formation. Beyond its role as a pro-atherogenic particle, oxidatively modified LDL also functions as a signalling molecule that dysregulates inflammatory responses, monocyte recruitment, smooth muscle cell proliferation, and endothelial apoptosis (Patel et al. 2000). The balance between NO and O₂⁻, regulated by superoxide dismutase, determines whether RNS/ROS interactions promote endothelial dysfunction or maintain vascular homoeostasis. Thus, the synergistic modification of LDL by ROS and RNS links oxidative stress to intracellular signalling pathways, driving atherosclerotic plaque initiation and progression (Gliozzi et al. 2019). This dysregulated redox environment amplifies oxidative stress, impairing endothelial nitric oxide bioavailability through nitrosative inactivation, thereby inducing endothelial dysfunction and promoting leucocyte adhesion (Madamanchi and Runge 2007). Mitochondrial ROS overproduction further exacerbates vascular injury by oxidising lipids, proteins, and mitochondrial DNA (mtDNA), destabilising cellular homoeostasis (Kattoor et al. 2017).

#### 2.2.2 Mitochondrial DNA damage in atherosclerosis

Human mtDNA is a circular, double-stranded molecule comprising 16,569 base pairs, with a light strand and a heavy strand, located within the mitochondrial matrix (Su et al. 2016). It replicates independently of the nuclear genome and encodes 37 essential genes, including 13 protein-coding genes that encode subunits of respiratory chain complexes I, III, IV, and V—critical for proton pumping and embedded in the inner mitochondrial membrane—as well as 2 ribosomal RNAs and 22 transfer RNAs necessary for mitochondrial protein synthesis (Kolesnikov and Gerasimov 2012; Wallace 2018). This underscores the dual role of mtDNA-encoded proteins in both energy-generating oxidative phosphorylation (OXPHOS) processes and mitochondrial translation (Chocron et al. 2019). However, mtDNA is particularly vulnerable to damage, mutations, and copy number variations due to its lack of histone protection, proximity to ROS production sites, and limited DNA repair capacity (Casoli et al. 2015; Wu et al. 2021). Recent studies have shown that mtDNA mutations are intricately linked to cardiovascular diseases through the modulation of systemic metabolic inflammation, proliferation, and apoptosis, contributing to atherosclerosis (Lemieux et al. 2011). Notably, mtDNA is released into the cytoplasm or circulation as a damage-associated molecular pattern (DAMP), triggering inflammatory pathways such as cGAS-STING, NOD-like receptor protein 3 (NLRP3) inflammasome, and TLR9 signalling, which exacerbate atherosclerosis progression (Zhang et al. 2025). NLRP3 inflammasomes are central to intracellular innate immunity and regulate inflammatory responses (Grebe et al. 2018). The activation of NLRP3 inflammasomes involves two pathways: the canonical pathway, which consists of priming (Signal 1) and activation (Signal 2), and a non-canonical pathway (Jo et al. 2016; Wang et al. 2023). The canonical pathway, relevant to atherosclerosis, operates via a two-step mechanism. In Signal 1 (priming), pattern recognition receptors (e.g. TLRs) or cytokine receptors activate NF-κB, promoting transcriptional upregulation of NLRP3 and pro-IL-1β/IL-18 (Jo et al. 2016). In Signal 2 (activation), various intracellular triggers—including K⁺ efflux, mitochondrial damage, ROS production, lysosomal rupture, and trans-Golgi dispersion—induce NLRP3 assembly (Tanase et al. 2023). This leads to caspase-1 activation, which cleaves pro-IL-1β and pro-IL-18 into mature cytokines, as well as gasdermin D (GSDMD) cleavage to trigger pyroptosis (Liaqat et al. 2020). Together, these events critically propagate vascular inflammation and contribute to atherosclerotic progression. MtDNA aberrations exert profound effects on three key cell types involved in atherogenesis: endothelial cells (ECs), macrophages, and VSMCs (Pie et al. 2018; Oh et al. 2020). In ECs, damaged mtDNA released into the cytoplasm triggers inflammatory cascades and accelerates endothelial dysfunction (Nakahira et al. 2015; Zhang et al. 2025). Xie et al. recently showed that gasdermin E induces mitochondrial membrane rupture in endothelial cells, leading to the release of mtDNA into the cytoplasm and the activation of the cGAS-STING pathway, exacerbating vascular inflammation and plaque progression (Xie et al. 2023). In macrophages, mtDNA release amplifies pro-inflammatory signalling, promoting foam cell formation and plaque expansion. Natarajan et al. revealed that the macrophage surface molecule VCAM-1 promotes mtDNA synthesis by activating the transcription factor C/EBPα, exacerbating inflammation and atherosclerosis (Natarajan et al. 2024). In VSMCs, mtDNA damage drives phenotypic switching, calcification, and apoptosis, destabilising atherosclerotic plaques (Nakahira et al. 2015; Zhang et al. 2025). Collectively, these findings highlight the pivotal role of mtDNA integrity in maintaining vascular homoeostasis and its pathological implications in cardiovascular diseases (Yu et al. 2013; Zhang et al. 2025).

#### 2.2.3 Mitophagy in atherosclerosis

Mitophagy, a selective degradation process for damaged mitochondria, plays a pivotal role in maintaining mitochondrial homoeostasis and reducing ROS production. The PINK1/Parkin-mediated pathway represents the most well-characterised mechanism of mitophagy (Lemasters 2005). Under physiological conditions, mitophagy efficiently eliminates dysfunctional mitochondria, preserving intracellular calcium homoeostasis, cellular signalling, and ATP synthesis while suppressing ROS levels and oxidative damage (Wu et al. 2022). Under stress conditions such as nutrient deprivation, mechanical injury, or ischaemia/hypoxia, defective mitophagy leads to the accumulation of dysfunctional mitochondria, resulting in excessive ROS generation. This exacerbates mitochondrial damage, triggering pro-apoptotic protein release into the cytoplasm, amplifying inflammatory responses, and causing EC injury. Concurrently, VSMC proliferation and phenotypic switching are promoted, while impaired mitophagy induces macrophage polarisation alterations and metabolic dysfunction, ultimately leading to cell death and unstable AS plaque rupture (Lin et al. 2022; Zhang et al. 2023). These findings underscore mitophagy dysfunction as a critical driver of AS pathogenesis, highlighting its potential as a therapeutic target to restore mitochondrial quality control and stabilise vulnerable plaques.

#### 2.2.4 Mitochondrial dynamics and biogenesis in atherosclerosis

Emerging evidence highlights the crucial role of mitochondrial homoeostasis—regulated by dynamic processes of fusion, fission, and biogenesis—in modulating key cellular events that drive atherosclerotic progression (Forte et al. 2021). Mitochondrial morphology and function are dynamically regulated by opposing processes of fusion and fission (Forte et al. 2021). Fusion, mediated by mitofusins (Mfn1, Mfn2) on the outer membrane and optic atrophy 1 on the inner membrane, facilitates the merging of mitochondrial contents, enabling complementation of damaged components such as mitochondrial DNA (mtDNA) and proteins (Yu and Bennett 2014). This process enhances oxidative phosphorylation, increases mitochondrial membrane potential, and promotes cellular metabolic efficiency. Conversely, fission, primarily executed by dynamin-related protein 1 (Drp1) and its mitochondrial receptor Fis1, segregates dysfunctional mitochondrial segments for targeted removal via mitophagy (Makino et al. 2010). Under physiological conditions, a balanced cycle of fusion and fission maintains a heterogeneous yet stable mitochondrial network, which is essential for energy production, calcium handling, and redox homoeostasis (Huynh and Heo 2021). In the context of atherosclerosis, however, this balance is significantly disrupted. Proatherogenic stimuli—including oxidative stress, inflammatory cytokines, and metabolic disturbances such as hyperlipidemia and hyperglycaemia—induce excessive mitochondrial fission while impairing fusion machinery. In vascular smooth muscle cells (VSMCs), increased Drp1 activity results in mitochondrial fragmentation, leading to bioenergetic deficits, heightened reactive oxygen species (ROS) production, and activation of apoptotic pathways (Huynh and Heo 2021). This not only compromises VSMC survival and collagen synthesis, weakening the protective fibrous cap of plaques, but also accelerates phenotypic switching and calcification. Similarly, in macrophages, fragmented mitochondria exhibit increased ROS emission and release of mitochondrial damage-associated molecular patterns (mtDAMPs), such as mtDNA and cardiolipin, which potentiate the activation of the NLRP3 inflammasome and promote interleukin-1β-mediated inflammation. Impaired fusion further reduces the ability to buffer mtDNA mutations, exacerbating mitochondrial dysfunction and fuelling a pro-inflammatory environment within the plaque (Huynh and Heo 2021). Mitochondrial biogenesis—the generation of new mitochondria—is orchestrated by the transcriptional coactivator peroxisome proliferator-activated receptor gamma coactivator 1-alpha (PGC-1α) and downstream factors, including nuclear respiratory factors and mitochondrial transcription factor A. This process is essential for adapting to energy demands and replacing damaged organelles (Forte et al. 2021). In atherosclerosis, however, biogenesis is often suppressed. In endothelial cells, reduced PGC-1α activity under conditions of disturbed flow or metabolic stress diminishes mitochondrial mass and oxidative capacity, impairing nitric oxide bioavailability and promoting a pro-adhesive, pro-inflammatory phenotype (Kadlec et al. 2016). In macrophages, compromised biogenesis limits ATP production, which is required for cholesterol efflux via transporters such as ABCA1 and ABCG1, thereby exacerbating foam cell formation and lipid retention within lesions (Kadlec et al. 2016). Dysregulated mitochondrial dynamics and biogenesis create a vicious cycle: fission dominance and fusion deficiency lead to mitochondrial fragmentation and dysfunction; decreased biogenesis limits regenerative capacity; and impaired mitophagy allows the accumulation of damaged organelles. Together, these alterations promote oxidative stress, inflammation, cellular senescence, and death—hallmarks of atherosclerotic plaque progression and vulnerability.

## 3 Foundations of mitochondrial-targeted delivery systems

Mitochondrial dysfunction is implicated in various human diseases, including cancer, cardiovascular disorders, and neurodegenerative conditions, making the targeted delivery of therapeutic molecules to mitochondria a critical challenge in molecular pharmacology. Although mitochondrial-targeting strategies were first developed nearly 50 years ago, their widespread application in drug delivery has significantly increased in the past decade (Zinovkin and Zamyatnin 2019). Several mitochondrial drug delivery systems (DDS) have been reported to date, encompassing antioxidant therapies, cancer treatments, mitochondrial gene therapy, and cell transplantation-based therapies, all utilising mitochondrial-targeted nanocarriers to achieve precise therapeutic outcomes (Yamada et al. 2020). Nanomedicine-mediated mitochondrial targeting offers significant advantages: nanocarriers (NPs) leverage their high surface area-to-volume ratio, small size, and ease of functionalization to enhance mitochondrial targeting efficiency while minimising systemic toxicity. Further refinement through ligand modifications improves delivery precision, while NPs mitigate therapeutic efficacy losses associated with traditional methods by optimising physical stability and drug solubility (Choudhary et al. 2021). Mitochondrial-targeted delivery requires hierarchical design strategies that align with mitochondrial biology and disease pathology. The first layer focuses on modulating specific mitochondrial functions (e.g. metabolic regulation); the second layer achieves suborganellar precision (e.g. membrane channels or matrix targeting); and the third layer integrates disease-specific phenotypes to enhance safety (Milane et al. 2015). Three primary mitochondrial-targeting mechanisms dominate: (1) accumulation of lipophilic cations, such as triphenylphosphonium (TPP+), driven by the mitochondrial membrane potential (ΔΨm); (2) binding of mitochondrial-penetrating peptides to cardiolipin (CL) on the inner mitochondrial membrane; and (3) import of nuclear-encoded proteins via TOM/TIM complexes, guided by N-terminal mitochondrial targeting sequences. These mechanisms exploit unique mitochondrial features—ΔΨm-dependent cation retention, CL-rich inner membrane domains, and conserved protein import pathways—which underlie the development of mitochondrial-targeted compounds, often enhanced through multi-strategy integration (Brown et al. 2017; Zinovkin and Zamyatnin 2019). Recent biotechnological advances have opened new avenues for directly modulating mitochondrial function and dynamics, offering transformative therapeutic strategies for mitochondrial-related diseases. These advances include a diverse range of tools: (1) novel nanocarrier platforms, such as polymeric nanoparticles, lipid-based systems, and inorganic nanoparticles; (2) molecular targeting ligands, like cell-penetrating peptides (CPPs), that enhance organelle specificity; and (3) gene-editing technologies for the precise manipulation of mitochondrial or nuclear genomes regulating mitochondrial biogenesis. The convergence of these technologies enables the development of precision nanomedicines capable of overcoming biological barriers and achieving subcellular targeting, thereby addressing the root causes of mitochondrial dysfunction in atherosclerosis.

## 4 Mitochondria-targeted delivery systems in atherosclerosis therapy

**Figure 2.**
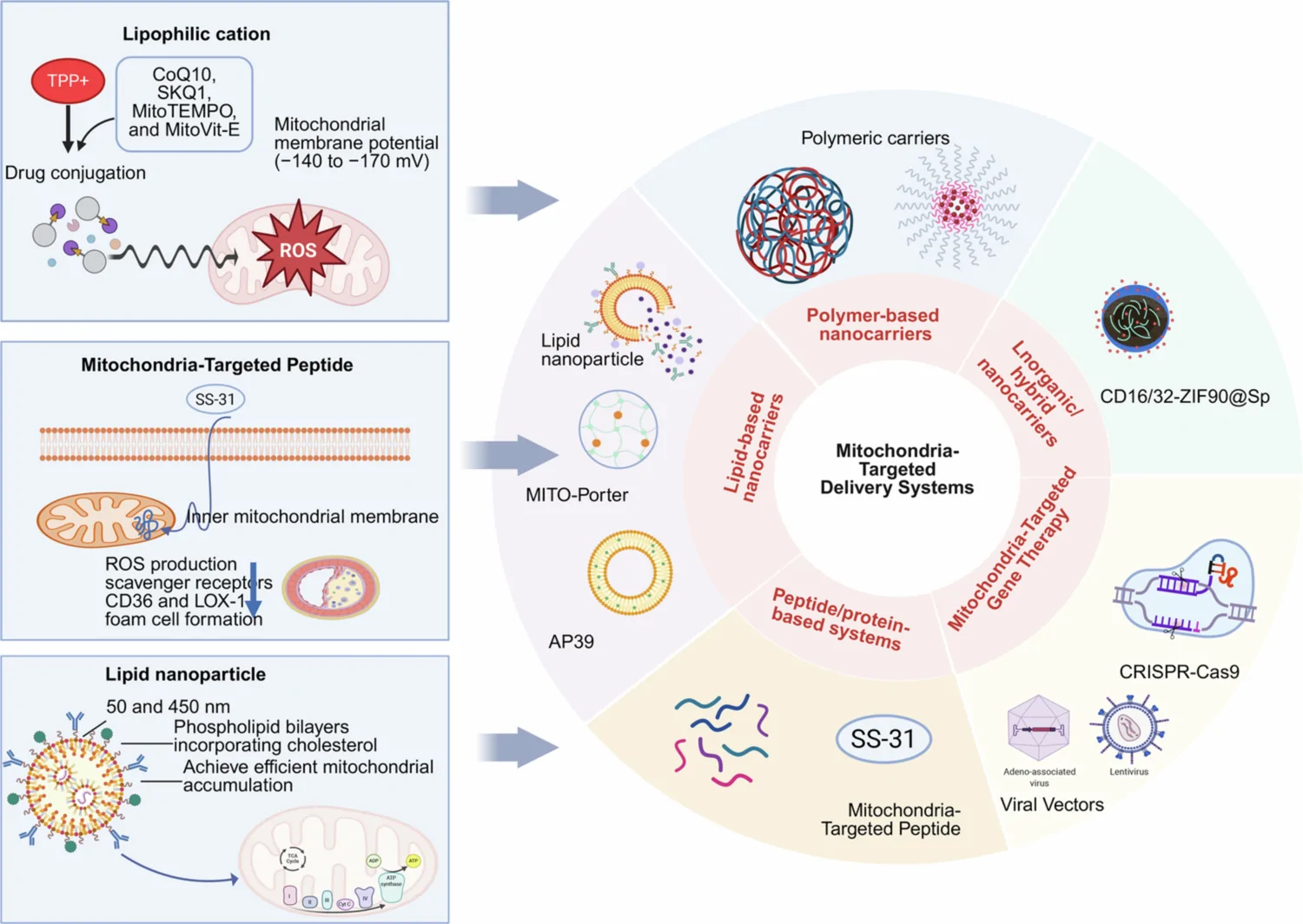
Overview and classification of mitochondria-targeted delivery systems for atherosclerosis therapy. This schematic outlines the principal categories of drug delivery systems engineered to target mitochondria within atherosclerotic plaques. Lipid-based carriers include TPP⁺-conjugated antioxidants (e.g. Mito), which exploit the mitochondrial membrane potential, and mucogenic MITO-Porter liposomes designed for macromolecular delivery. Polymer-based nanoparticles, such as PLGA, enable controlled release of therapeutic agents, while inorganic systems—including antibody-targeted ZIF-90 nanoparticles—facilitate cell-specific mitochondrial targeting. Peptide-based strategies involve mitochondria-homing peptides (e.g. SS-31) and cell-penetrating peptides. Additionally, gene-editing technologies, such as CRISPR-Cas9, represent advanced approaches for correcting mitochondrial DNA mutations.

An overview of the major classes of mitochondria-targeted delivery systems explored for AS therapy is provided in Figure 2.

### 4.1 Classification and design principles of mitochondrial-targeted delivery systems

The efficient delivery of therapeutic agents to mitochondria, subcellular organelles surrounded by a double membrane, remains a significant challenge in nanomedicine. To overcome various biological barriers—including systemic circulation, extravasation at atherosclerotic plaques, cellular uptake, endolysosomal escape, and translocation across the mitochondrial membranes—mitochondria-targeted nanocarriers must be meticulously engineered. These carriers can be classified based on their core material composition and primary targeting mechanism, with their design governed by fundamental physicochemical principles that dictate their in vivo behaviour. Mitochondrial nanocarriers are primarily constructed from several material categories, each offering unique advantages.

### 4.2 Lipid-based nanocarriers

#### 4.2.1 TPP⁺-modified liposomes (and lipophilic cation conjugates)

Liposomes functionalized with mitochondriotropic ligands, particularly TPP⁺, are pivotal in mitochondrial-targeted therapies due to their biocompatibility and versatile loading capacity (Zielonka et al. 2017). TPP⁺ takes advantage of the high negative mitochondrial membrane potential (ΔΨm) for electrophoretic accumulation within the organelle, a widely used strategy for delivering antioxidants to counteract mitochondrial reactive oxygen species (mtROS), a key contributor to AS (Zielonka et al. 2017).

TPP⁺, an amphiphilic delocalised cation, selectively accumulates in mitochondria due to the high negative mitochondrial membrane potential (−140 to −170 mV) when conjugated to lipophilic hydrocarbon chains (Wang et al. 2020). Notable TPP-based antioxidants include MitoQ, SKQ1, MitoTEMPO, and MitoVit-E.

MitoQ, a conjugate of TPP⁺ and coenzyme Q10 (CoQ10)—a critical antioxidant and cofactor in mitochondrial oxidative phosphorylation (Rodriguez-Cuenca et al. 2010; Sacks et al. 2021)—penetrates phospholipid bilayers and accumulates at the inner mitochondrial membrane's matrix-facing surface. This effectively reduces ROS, mitigates mtDNA damage, and attenuates atherosclerosis progression (Li et al. 2014; Rossman et al. 2018). Animal studies have confirmed CoQ10's ability to inhibit endothelial inflammation, improve endothelial lipid metabolism, and reduce thrombosis. CoQ10 has been shown to inhibit arterial lipid oxidation in experimental rabbits, reduce mitochondrial oxidative stress, and lessen atherosclerotic plaque formation in mice (Natarajan et al. 2024; Liao et al. 2024). Clinical studies indicate that a Mediterranean diet combined with CoQ10 supplementation can reduce the expression of oxidative stress-related genes and proteins and activate p53-dependent DNA repair pathways, suggesting long-term preventive potential for arteriosclerosis (Gutierrez-Mariscal et al. 2014). However, due to the limited sample size and short follow-up period, further validation is required. Similarly, SKQ1, a mitochondrial-targeted derivative of plastoquinone, selectively accumulates in the mitochondrial matrix of cardiomyocytes and endothelial cells via electrochemical gradients (Jia et al. 2025). It inhibits mitochondrial ROS, reduces mtDNA oxidation, and prevents NLRP3 inflammasome activation by disrupting the ox-mtDNA-NLRP3 interaction, demonstrating effective anti-inflammatory effects. Despite its promising cardiac protection, additional studies, including in animal models like ApoE⁻/⁻ mice, are needed to assess its impact on plaques (Fedorov et al. 2022; Jia et al. 2025). Furthermore, MitoVit-E, a TPP-conjugated vitamin E analogue, accumulates 100- to 1,000-fold in mitochondria, suppresses VEGF-induced mtROS production, inhibits PAK/Akt/p38MAPK/ERK1/2 phosphorylation, and reduces endothelial migration, thereby alleviating oxidative damage and atherosclerosis (Wang et al. 2011; Bhatti et al. 2017). Recent advancements include the development of Mito-Esc, a mitochondria-targeted esculetin derivative modified with octyl-TPP⁺. Mito-Esc ameliorates hyperglycaemia-induced atherosclerosis in db/db mice by restoring redox balance through the mitochondrial-metabolic-oxidative stress network, activating the AMPK-eNOS axis, and elevating SIRT1 levels to reduce vascular inflammation and endothelial dysfunction (Singuru et al. 2024). Additionally, it can inhibit macrophage polarisation, reduce pro-inflammatory factor release, suppress VSMC migration and proliferation, resist platelet aggregation, and prevent LDL and HDL oxidation (Wang et al. 2022). Moreover, combined Mito-Esc and metformin therapy enhances fatty acid *β*-oxidation (FAO) in endothelial cells, activates the AMPK-SIRT3 pathway, boosts nitric oxide production, and inhibits Ang-II-induced plaque formation in Apoe⁻/⁻ mice, demonstrating its potential as a precision mitochondrial therapy for metabolic cardiovascular diseases (Pulipaka et al. 2024; Pulipaka et al. 2024). The preferential mitochondrial accumulation of these TPP-linked compounds, driven by the high membrane potential, underscores their efficacy in targeting mitochondrial pathologies (Pulipaka et al. 2024). Esculetin, a pleiotropic natural compound, has demonstrated unique potential in targeted atherosclerosis therapy through animal experiments. However, its clinical application remains limited due to pharmacokinetic deficiencies and insufficient mechanistic research. Future advancements, such as improved formulations and interdisciplinary collaborations (e.g. pharmacy, molecular biology, and clinical medicine), are necessary to overcome bioavailability challenges and identify core molecular targets. With these developments, esculetin may become a promising option for comprehensive atherosclerosis management, particularly for early lesion intervention and combined treatment strategies.

Beyond small-molecule conjugates, TPP⁺ can also be integrated into liposomal bilayers to create targeted carriers. For example, the *β*-MEND system utilises a TPP⁺-modified liposome to deliver resveratrol to cardiomyocyte mitochondria, enhancing respiratory capacity (Tsujioka et al. 2021). These examples highlight TPP⁺-modified liposomes as a versatile platform for delivering a range of antioxidant compounds with high mitochondrial specificity.

Beyond antioxidants, the TPP⁺ strategy has been successfully applied to deliver other therapeutic agents. A notable example is AP39, a mitochondria-targeted hydrogen sulphide (H₂S) donor, where an H₂S-releasing moiety is conjugated to TPP⁺ (Szczesny et al. 2014). AP39 exploits the ΔΨm for mitochondrial accumulation and has been shown to favourably modulate the plaque microenvironment. In preclinical studies, AP39 administration shifted macrophage polarisation from a pro-inflammatory M1-like phenotype to an anti-inflammatory M2-like state within atherosclerotic plaques and upregulated uncoupling protein 1 (UCP1) expression in VSMCs, collectively attenuating atherogenesis (Szczesny et al. 2014; Stachowicz et al. 2024). These findings underscore the potential of TPP⁺-mediated delivery to precisely administer gasotransmitters such as H₂S for metabolic-immune regulation in AS. However, clinical translation requires further validation regarding long-term safety and optimal dosing (Szczesny et al. 2014; Stachowicz et al. 2024).

#### 4.2.2 Other lipid-based systems

In addition to TPP⁺, other lipid molecules possess intrinsic mitochondrial affinity. For instance, 1,10-decamethylene bis-4-aminoquinaldinium chloride (Dequalinium, DQA) self-assembles into vesicular structures that can encapsulate drugs and preferentially localise to mitochondria (Zielonka et al. 2017). Furthermore, advanced fusogenic lipid nanocarriers utilise membrane-active components to overcome delivery barriers. One prominent example is the MITO-Porter system, a lipid-based nanocapsule functionalized with the cell-penetrating peptide octa-arginine (R8). MITO-Porter enables efficient cytoplasmic and mitochondrial delivery of macromolecules via membrane fusion (Yamada et al. 2008; Buchke et al. 2022; Sato et al. 2024). Mitsue Hibino et al. developed a mitochondria-targeted nanocapsule (MITO-Porter) functionalized with R8 to encapsulate coenzyme Q10 (Hibino et al. 2025). The targeting mechanism is based on liposomes carrying R8 membrane-penetrating peptides, which interact electrostatically with the negatively charged mitochondrial membrane, facilitating membrane fusion and overcoming the mitochondrial double-membrane barrier (Hibino et al. 2025). Experimental results have demonstrated that this drug exhibits both antioxidative and metabolic regulation properties in mouse macrophages, solving CoQ10’s low bioavailability problem, enhancing R8-mediated targeting efficiency, and synergistically modulating macrophage immune responses. These findings support MITO-Porter's efficacy in vivo, though direct animal experimental data were not provided in the study. Future research should expand to include different disease models.

### 4.3 Polymer-based nanocarriers

Polymeric carriers, such as poly(lactic-co-glycolic acid) (PLGA) nanoparticles, offer controlled release and high cargo loading. When functionalized with TPP⁺, PLGA nanoparticles have been used to deliver antioxidants like resveratrol to endothelial cell mitochondria, enhancing anti-inflammatory effects compared to free drugs (Li et al. 2024). Similarly, polymeric micelles self-assembled from amphiphilic block copolymers conjugated with mitochondrial targeting peptides show promise in co-delivering therapeutic agents to plaque macrophages (Szeto 2006).

### 4.4 Inorganic and hybrid nanocarriers

Recently, Chen et al. engineered a CD16/32 antibody-modified zinc-based metal-organic framework (MOF) nanoparticle, ZIF90, for targeted spermine (Sp) delivery to intraplaque macrophages (Chen et al. 2025). The ZIF90-Sp complex exhibits mitochondrial tropism, releasing Sp upon interaction with mitochondrial ATP (Jiang et al. 2019). CD16/32 modification enhances specificity for pro-inflammatory macrophages, while the system stabilises mitochondrial membrane potential, restores respiratory chain activity, and inhibits ferroptosis by reducing lipid peroxidation and upregulating GPX4/xCT expression (Zhou et al. 2020; Chen et al. 2025). Both in vitro and in vivo experiments have demonstrated that CD16/32-ZIF90@Sp can precisely target macrophages and oxidised low-density lipoprotein (OX-LDL)-activated macrophages within atherosclerotic plaques (Chen et al. 2025). Furthermore, CD16/32-ZIF90@Sp treatment effectively mitigated the progression of AS and iron-related cell death in plaque macrophages of ApoE-deficient mice without causing significant side effects. Compared with systemic Sp administration, ZIF90-Sp enhances mitochondrial targeting efficiency, minimises toxicity risks, and represents a precise nanoplatform that combines multi-mechanism synergy, biosafety, and targeted efficacy. Although this nanoparticle shows promise in precisely and effectively targeting macrophages, translating this success into clinical applications remains challenging.

### 4.5 Peptide and protein-based delivery systems

#### 4.5.1 Mitochondria-targeting peptides

Peptides are a unique class of mitochondriotropic vectors. Szeto-Schiller (SS) peptides, a subclass of mitochondrial-targeting molecules, are small, permeable, and accumulate specifically in mitochondria (Zhang et al. 2021). Typically composed of three to five amino acids with alternating aromatic and basic residues, SS peptides localise to the inner mitochondrial membrane, where they reduce intracellular reactive oxygen species (ROS), preserve membrane potential, and inhibit lipid peroxidation, thereby mitigating mitochondrial dysfunction (Szeto 2006; Li et al. 2025). Among these, the tetrapeptide SS-31 (elamipretide) is the most extensively studied. In mouse models, Zhang et al. found that SS-31 treatment significantly suppresses ROS production, enhances superoxide dismutase activity, and inhibits the upregulation of scavenger receptors CD36 and LOX-1, thereby attenuating foam cell formation (Zhang et al. 2017; Shan et al. 2023; Long et al. 2024). In vivo studies of atherosclerosis further suggest that SS-31 delays plaque progression by regulating the mitochondrial-inflammatory axis (Xu et al. 2023). However, clinical translation of SS-31 remains limited due to challenges such as inefficient targeted delivery, long-term safety concerns, and the need for human pharmacokinetic data. Future research should focus on integrating nanocarrier technology to optimise bioavailability and conducting multi-centre clinical trials to assess its impact on cardiovascular outcomes, thereby advancing its potential as a strategy for precise mitochondrial treatment of.

#### 4.5.2 Cell-penetrating peptides as mitochondrial vectors

Cell-penetrating peptides (CPPs) are short, non-toxic peptides with cationic and amphipathic properties, allowing them to traverse cellular membranes (Guidotti et al. 2017; Zorko and Langel 2022). These peptides are used to deliver various cargoes, including proteins, oligonucleotides, and therapeutic molecules (Cerrato et al. 2015).

### 4.6 Mitochondria-targeted gene therapy

Mitochondrial dysfunction due to mtDNA damage contributes to cellular injury and death in atherosclerosis (An et al. 2023). As a result, dysfunctional mtDNA has emerged as a promising target for therapeutic strategies in atherosclerosis (Liu et al. 2025). Recent advances in mitochondrial disease therapy include the direct delivery of nucleic acids into mitochondria. Engineered nanocarriers for mitochondrial DNA delivery have demonstrated significant efficacy with low cytotoxicity (Zakirov et al. 2020; Liu et al. 2025). Studies of ruptured plaques, arterial intima, and blood samples indicate that mutations or damage in mitochondrial genes—such as those encoding ETC proteins (e.g. NADH dehydrogenase, ATP synthase, cytochrome b, cytochrome c oxidase subunits, and tRNA genes)—are associated with cellular injury and atherogenesis (Wongrakpanich et al. 2014; Forrester and Griendling 2017; Zakirov et al. 2020). Transfection of these genes may reduce plaque progression and attenuate atherosclerotic lesion development. Gene therapy offers the potential to directly repair or replace damaged mtDNA using tools like CRISPR-Cas9 (Goncalves and Paiva 2017). Additionally, mitochondrial transfer and RNA interference technologies are being explored to restore mitochondrial integrity. Current strategies include viral vectors, CRISPR/Cas9 systems, and stem cell-based therapies (Gammage et al. 2018; Hussain et al. 2021; Zhou and Wang, 2025). These approaches aim to address mtDNA-related abnormalities in atherosclerosis, with future advancements poised to establish a comprehensive framework for targeted mitochondrial genome editing. Together, these innovations highlight the transformative potential of mitochondrial-targeted gene therapy in combating atherosclerosis and related cardiovascular diseases. Key examples of mitochondrial-targeted nanotherapeutics and their mechanisms are summarised in Table 1.

**Table 1.** Several mitochondrial-targeted drugs for anti-atherosclerosis.

Nanoparticle	Cargo	Cell type	Mechanism and effect	References
TPP	CoQ10	Endothelial cell	Upregulate mitophagy Modulate the NF-κB pathway Reduce leucocyte-endothelial interactions	Gutierrez-Mariscal et al. (2014); Liao et al. (2024)
TPP	SKQ1	Endothelial cell Macrophage Cardiomyocyte	Inhibit mitochondrial ROS Reduce mtDNA oxidation Block NLRP3 inflammasome activation	Jia et al. (2025)
TPP	vitamin E	Endothelial cell	Suppress VEGF-induced mtROS production Inhibit PAK/Akt/p38MAP/ERK1/2 phosphorylation	Wang et al. (2011); Bhatti et al. (2017)
TPP	Esculetin	Endothelial cell Macrophage Smooth muscle cells	Activate AMPK-eNOS axis Elevate SIRT1 levels Alter macrophage polarisation Inhibit pro-inflammatory cytokine	Singuru et al. (2024)
*β*-MEND	RES	Cardiomyocyte	Enhance maximal respiratory capacity in cardiomyocytes Maintain cell viability	Machado et al. (2019)
TPP	SS-31 astaxanthin	Endothelial cell Macrophage Smooth muscle cells	Suppress ROS production Enhance superoxide dismutase activity Attenuate foam cell formation	Xu et al. (2023)
TPP	AP39	Macrophage Smooth muscle cells	Change macrophage phenotypes Increase UCP1 expression in vascular smooth muscle cells	Stachowicz et al. (2024)
R8 (+) MITO-Porter	CoQ10	Macrophage	Inhibit the differentiation of proinflammatory macrophage differentiation	Hibino et al. (2025)
ZIF90	Sp	Macrophage	Attenuate the ferroptosis of macrophage	Hibino et al. (2025)

## 5 Discussion

The mitochondrial-targeted delivery system, a cutting-edge approach for the precise treatment of atherosclerosis, has shown significant progress. Mitochondria-targeted platforms—such as liposomes, polymeric nanoparticles, and peptide-based carriers—can selectively deliver antioxidants (e.g. MitoQ, SS-31), mitophagy inducers, or genetic regulators to mitochondria. This selective delivery directly scavenges reactive oxygen species (ROS), inhibits NLRP3 inflammasome assembly, and restores mitochondrial homoeostasis, thereby disrupting the ‘mitochondrial damage-inflammation-plaque instability’ cycle (Pathak et al. 2015). Notably, SS-31, which has advanced to Phase II clinical trials, improves endothelial function and inhibits macrophage foam cell formation by targeting cardiolipin, while MitoQ alleviates vascular inflammation by enhancing nitric oxide bioavailability. Xu et al. demonstrated that the PA/ASePSD nanoplatform effectively combines SS-31 with astaxanthin, along with photoacoustic imaging, offering a non-invasive real-time method for diagnosing atherosclerosis and simultaneously inhibiting plaque formation (Xu et al. 2023). Both compounds exhibit substantial potential for clinical translation. Additionally, multimodal nanocarriers, including biomimetic membrane-coated liposomes and polymer nanoparticles, have achieved synergistic intervention in AS-related oxidative stress, lipid accumulation, and immune dysregulation through co-delivery of antioxidants and metabolic regulators.

Currently, the targeted delivery of nanocarriers must overcome four major biological barriers: blood circulation stability, tissue penetration, transmembrane delivery, and lysosomal escape (Li et al. 2024). Several challenges remain in mitochondrial-targeted delivery. First, the design of nanocarriers must address the issue of low drug delivery efficiency. To improve drug loading efficiency, amphiphilic small molecules can be used for self-assembly. Coupling hydrophilic cations with hydrophobic drugs to form amphiphilic drug derivatives can enhance stability, circulation time, and tissue accumulation (Long et al. 2024). Second, premature drug release, which can reduce therapeutic efficacy or increase side effects, must be addressed. Developing responsive release mechanisms to enable precise drug delivery in specific environments, such as low pH or high ROS conditions, is an essential area for future research. Some current strategies, such as the use of ΔΨm-responsive materials and pH-dependent responsive materials, may offer partial solutions to this issue. Additionally, mitochondrial-targeted peptides face challenges such as large molecular size, poor solubility, and limited cell membrane permeability (Yousif et al. 2009). Designing customisable, multifunctional delivery systems that meet the demands of precise mitochondrial targeting is a promising direction for future research. Lastly, although many nanomaterials demonstrate significant effects in animal models, they may encounter issues such as insufficient efficacy, safety concerns, or low response rates due to individual variability in clinical trials. In the future, through innovations in targeted strategies, biomaterial optimisation, precise treatment stratification, and advancements in clinical translation technologies, the bottlenecks of nanomedicines in atherosclerosis treatment can be systematically addressed.

Interdisciplinary integration, such as CRISPR-Cas9-assisted mitochondrial genome editing and AI-driven vector optimisation, will be crucial for the development of bionic intelligent delivery systems (Hussain et al. 2021). These systems, in combination with multi-level targeting strategies (e.g. cyclic targeting and mitochondrial localisation) and in situ synthesis technologies, aim to achieve on-demand drug release and sustained regulation within plaque mitochondria. These breakthroughs will enable a shift from organ-level interventions to subcellular precision medicine, providing innovative solutions for metabolic cardiovascular diseases.

## 6 Conclusions

In this study, mitochondrial-targeted nanomaterials were shown to delay the progression of atherosclerotic plaques by precisely delivering cellular mitochondria. Future advancements in the optimisation of targeted ligands and the design of responsive carriers are expected to enable dynamic regulation of the plaque microenvironment, presenting an innovative strategy for combined treatment and precise intervention in atherosclerosis.

## Data Availability

Data sharing is not applicable to this article as no new data were created or analysed in this research.

## References

[cit0001] Alevriadou BR, Shanmughapriya S, Patel A et al. 2017. Mitochondrial Ca(2+) transport in the endothelium: regulation by ions, redox signalling and mechanical forces. J R Soc Interface. 14(137):20170672. 10.1098/rsif.2017.0672 29237825 PMC5746573

[cit0002] An C, Sun F, Liu C et al. 2023. IQGAP1 promotes mitochondrial damage and activation of the mtDNA sensor cGAS-STING pathway to induce endothelial cell pyroptosis leading to atherosclerosis. Int Immunopharmacol. 123:110795. 10.1016/j.intimp.2023.110795 37597406

[cit0003] Bhatti JS, Bhatti GK, Reddy PH. 2017. Mitochondrial dysfunction and oxidative stress in metabolic disorders - A step towards mitochondria based therapeutic strategies, biochimica et biophysica acta. Molecular basis of disease. 1863(5):1066–1077. 10.1016/j.bbadis.2016.11.010 27836629 PMC5423868

[cit0004] Brown DA, Perry JB, Allen ME et al. 2017. Expert consensus document: mitochondrial function as a therapeutic target in heart failure. Nat Rev Cardiol. 14(4):238–250. 10.1038/nrcardio.2016.203 28004807 PMC5350035

[cit0005] Buchke S, Sharma M, Bora A et al. 2022. Mitochondria-targeted, nanoparticle-based drug-delivery systems: therapeutics for mitochondrial disorders. Life (Basel). 12(5):657. 10.3390/life12050657 35629325 PMC9144057

[cit0006] Casoli T, Spazzafumo L, Di Stefano G et al. 2015. Role of diffuse low-level heteroplasmy of mitochondrial DNA in Alzheimer's disease neurodegeneration. Front Aging Neurosci. 7:142. 10.3389/fnagi.2015.00142 26257647 PMC4511837

[cit0007] Cerrato CP, Pirisinu M, Vlachos EN et al. 2015. Novel cell-penetrating peptide targeting mitochondria. FASEB J. 29(11):4589–4599. 10.1096/fj.14-269225 26195590

[cit0008] Chen Y, Xu B, Lin Q et al. 2025. Spermine delivered by ZIF90 nanoparticles alleviates atherosclerosis by targeted inhibition of macrophage ferroptosis in plaque. J Nanobiotechnology. 23(1):165. 10.1186/s12951-025-03271-8 40038689 PMC11877808

[cit0009] Cheng J, Huang H, Chen Y et al. 2023. Nanomedicine for diagnosis and treatment of atherosclerosis. Advanced science (Weinheim, Baden-Wurttemberg, Germany). 10(36):e2304294. 10.1002/advs.202304294 37897322 PMC10754137

[cit0010] Chocron ES, Munkácsy E, Pickering AM. 2019. Cause or casualty: the role of mitochondrial DNA in aging and age-associated disease, biochimica et biophysica acta. Molecular basis of disease. 1865(2):285–297. 10.1016/j.bbadis.2018.09.035 30419337 PMC6310633

[cit0011] Choudhary D, Goykar H, Karanwad T et al. 2021. An understanding of mitochondria and its role in targeting nanocarriers for diagnosis and treatment of cancer. Asian J Pharm Sci. 16(4):397–418. 10.1016/j.ajps.2020.10.002 34703491 PMC8520044

[cit0012] Du H, Xu T, Yu S et al. 2025. Mitochondrial metabolism and cancer therapeutic innovation. Signal Transduct Target Ther. 10(1):245. 10.1038/s41392-025-02311-x 40754534 PMC12319113

[cit0013] Falk E. 2006. Pathogenesis of atherosclerosis. J Am Coll Cardiol. 47(8 Suppl):C7–12. 10.1016/j.jacc.2005.09.068 16631513

[cit0014] Fedorov AV, Chelombitko MA, Chernyavskij DA et al. 2022. Mitochondria-targeted antioxidant SkQ1 prevents the development of experimental colitis in mice and impairment of the barrier function of the intestinal epithelium. Cells. 11(21):3441. 10.3390/cells11213441 36359839 PMC9659222

[cit0015] Forrester SJ, Griendling KK. 2017. Mitochondrial respiration and atherosclerosis: R-E-S-P-I-R-E. Find out what it means to mvarphi (and VSMC). Arterioscler Thromb Vasc Biol. 37(12):2229–2230. 10.1161/ATVBAHA.117.310298 29162598 PMC5785938

[cit0016] Forte M, Schirone L, Ameri P et al. 2021. Society of cardiology working group on, H. Molecular biology of the, the role of mitochondrial dynamics in cardiovascular diseases. Br J Pharmacol. 178(10):2060–2076. 10.1111/bph.15068 32294237

[cit0017] Gammage PA, Moraes CT, Minczuk M. 2018. Mitochondrial genome engineering: the revolution May not be CRISPR-Ized. Trends Genet. 34(2):101–110. 10.1016/j.tig.2017.11.001 29179920 PMC5783712

[cit0018] Gliozzi M, Scicchitano M, Bosco F et al. 2019. Modulation of nitric oxide synthases by oxidized LDLs: role in vascular inflammation and atherosclerosis development. Int J Mol Sci. 20(13):3294. 10.3390/ijms20133294 31277498 PMC6651385

[cit0019] Goncalves GAR, Paiva RMA. 2017. Gene therapy: advances, challenges and perspectives. Einstein (Sao Paulo). 15(3):369–375. 10.1590/S1679-45082017RB4024 29091160 PMC5823056

[cit0020] Grebe A, Hoss F, Latz E. 2018. NLRP3 inflammasome and the IL-1 pathway in atherosclerosis. Circ Res. 122(12):1722–1740. 10.1161/CIRCRESAHA.118.311362 29880500

[cit0021] Guidotti G, Brambilla L, Rossi D. 2017. Cell-penetrating peptides: from basic research to clinics. Trends Pharmacol Sci. 38(4):406–424. 10.1016/j.tips.2017.01.003 28209404

[cit0022] Gutierrez-Mariscal FM, Yubero-Serrano EM, Rangel-Zuniga OA et al. 2014. Postprandial activation of p53-dependent DNA repair is modified by Mediterranean diet supplemented with coenzyme Q10 in elderly subjects. J Gerontol A Biol Sci Med Sci. 69(7):886–893. 10.1093/gerona/glt174 24158762

[cit0023] Hibino M, Filosi T, Carrion LL et al. 2025. An effective approach to modulate mitochondrial function in murine primary macrophages by a mitochondria-targeted nanocapsule, MITO-Porter. Biomedicine & pharmacotherapy = Biomedecine & pharmacotherapie. 186:118019. 10.1016/j.biopha.2025.118019 40184839

[cit0024] Hussain SA, Yalvac ME, Khoo B et al. 2021. Adapting CRISPR/Cas9 system for targeting mitochondrial genome. Front Genet. 12:627050. 10.3389/fgene.2021.627050 33889176 PMC8055930

[cit0025] Huynh DTN, Heo KS. 2021. Role of mitochondrial dynamics and mitophagy of vascular smooth muscle cell proliferation and migration in progression of atherosclerosis. Arch Pharm Res. 44(12):1051–1061. 10.1007/s12272-021-01360-4 34743301

[cit0026] Jia H, Song Y, Hua Y et al. 2025. Molecular mechanism of aerobic exercise ameliorating myocardial mitochondrial injury in mice with heart failure. Int J Mol Sci. 26(5):2136. 10.3390/ijms26052136 40076760 PMC11901053

[cit0027] Jiang Z, Wang Y, Sun L et al. 2019. Dual ATP and pH responsive ZIF-90 nanosystem with favorable biocompatibility and facile post-modification improves therapeutic outcomes of triple negative breast cancer in vivo. Biomaterials. 197:41–50. 10.1016/j.biomaterials.2019.01.001 30640136

[cit0028] Jo EK, Kim JK, Shin DM et al. 2016. Molecular mechanisms regulating NLRP3 inflammasome activation. Cell Mol Immunol. 13(2):148–159. 10.1038/cmi.2015.95 26549800 PMC4786634

[cit0029] Kadlec AO, Chabowski DS, Ait-Aissa K et al. 2016. Role of PGC-1alpha in vascular regulation: implications for atherosclerosis. Arterioscler Thromb Vasc Biol. 36(8):1467–1474. 10.1161/ATVBAHA.116.307123 27312223 PMC4965312

[cit0030] Kattoor AJ, Pothineni NVK, Palagiri D et al. 2017. Oxidative stress in atherosclerosis. Curr Atheroscler Rep. 19(11):42. 10.1007/s11883-017-0678-6 28921056

[cit0031] Kolesnikov AA, Gerasimov ES. 2012. Diversity of mitochondrial genome organization. Biochemistry (Mosc. 77(13):1424–1435. 10.1134/S0006297912130020 23379519

[cit0032] Lemasters JJ. 2005. Selective mitochondrial autophagy, or mitophagy, as a targeted defense against oxidative stress, mitochondrial dysfunction, and aging. Rejuvenation Res. 8(1):3–5. 10.1089/rej.2005.8.3 15798367

[cit0033] Lemieux H, Semsroth S, Antretter H et al. 2011. Mitochondrial respiratory control and early defects of oxidative phosphorylation in the failing human heart. Int J Biochem Cell Biol. 43(12):1729–1738. 10.1016/j.biocel.2011.08.008 21871578

[cit0034] Li H, Horke S, Forstermann U. 2014. Vascular oxidative stress, nitric oxide and atherosclerosis. Atherosclerosis. 237(1):208–219. 10.1016/j.atherosclerosis.2014.09.001 25244505

[cit0035] Li Y, Li XM, Wei LS et al. 2024. Advancements in mitochondrial-targeted nanotherapeutics: overcoming biological obstacles and optimizing drug delivery. Front Immunol. 15:1451989. 10.3389/fimmu.2024.1451989 39483479 PMC11524880

[cit0036] Li J, Lan T, Guo Q et al. 2024. Mitochondria-targeted natural antioxidant nanosystem for diabetic vascular calcification therapy. Biomacromolecules. 25(7):4329–4343. 10.1021/acs.biomac.4c00375 38833553

[cit0037] Li Z, Wang H, Liu N et al. 2025. Renal lipid alterations from diabetes to early-stage diabetic kidney disease and mitophagy: focus on cardiolipin. J Cell Mol Med. 29(3):e70419. 10.1111/jcmm.70419 39936909 PMC11816159

[cit0038] Liao M, He X, Zhou Y et al. 2024. Coenzyme Q10 in atherosclerosis. Eur J Pharmacol. 970:176481. 10.1016/j.ejphar.2024.176481 38493916

[cit0039] Liaqat A, Asad M, Shoukat F et al. 2020. A spotlight on the underlying activation mechanisms of the NLRP3 inflammasome and its role in atherosclerosis: A review. Inflammation. 43(6):2011–2020. 10.1007/s10753-020-01290-1 32656610

[cit0040] Libby P, Buring JE, Badimon L et al. 2019. Atherosclerosis. Nat Rev Dis Primers. 5(1):56. 10.1038/s41572-019-0106-z 31420554

[cit0041] Lin J, Duan J, Wang Q et al. 2022. Mitochondrial dynamics and mitophagy in cardiometabolic disease. Front Cardiovasc Med. 9:917135. 10.3389/fcvm.2022.917135 35783853 PMC9247260

[cit0042] Liu X, Wang X, Li J et al. 2025. Cabbage exosome-like nanoparticles encapsulating small noncoding tsRNA prevent postinjury arterial restenosis. Research (Wash D C). 8:1019. 10.34133/research.1019 41311465 PMC12648583

[cit0043] Liu Y, Yi B, Yang L et al. 2025. Anisotropic Micro/Nanotopography regulating mitochondrial dynamics in cardiomyocytes. Research (Wash D C). 2025:0891. 10.34133/research.0891 40963698 PMC12439428

[cit0044] Liu Z, Huang N, Liu C et al. 2025. Mitochondrial DNA in atherosclerosis research progress: a mini review. Front Immunol. 16:1526390. 10.3389/fimmu.2025.1526390 39991161 PMC11842404

[cit0045] Long X, Liu M, Nan Y et al. 2024. Revitalizing ancient mitochondria with nano-strategies: mitochondria-remedying nanodrugs concentrate on disease control. Adv Mater. 36(18):e2308239. 10.1002/adma.202308239 38224339

[cit0046] Machado ND, Fernandez MA, Diaz DD. 2019. Recent strategies in resveratrol delivery systems. ChemPlusChem. 84(7):951–973. 10.1002/cplu.201900267 31943987

[cit0047] Madamanchi NR, Runge MS. 2007. Mitochondrial dysfunction in atherosclerosis. Circ Res. 100(4):460–473. 10.1161/01.RES.0000258450.44413.96 17332437

[cit0048] Makino A, Scott BT, Dillmann WH. 2010. Mitochondrial fragmentation and superoxide anion production in coronary endothelial cells from a mouse model of type 1 diabetes. Diabetologia. 53(8):1783–1794. 10.1007/s00125-010-1770-4 20461356 PMC2892085

[cit0049] Milane L, Trivedi M, Singh A et al. 2015. Mitochondrial biology, targets, and drug delivery. J Control Release. 207:40–58. 10.1016/j.jconrel.2015.03.036 25841699

[cit0050] Nakahira K, Hisata S, Choi AM. 2015. The roles of mitochondrial damage-associated molecular patterns in diseases. Antioxid Redox Signal. 23(17):1329–1350. 10.1089/ars.2015.6407 26067258 PMC4685486

[cit0051] Natarajan N, Florentin J, Johny E et al. 2024. Aberrant mitochondrial DNA synthesis in macrophages exacerbates inflammation and atherosclerosis. Nat Commun. 15(1):7337. 10.1038/s41467-024-51780-1 39187565 PMC11347661

[cit0052] Oh S, Son M, Park CH et al. 2020. The reducing effects of Pyrogallol-Phloroglucinol-6,6-Bieckol on high-fat diet-induced pyroptosis in endothelial and vascular smooth muscle cells of mice aortas. Mar Drugs. 18(12):648. 10.3390/md18120648 33339328 PMC7766911

[cit0053] Orekhov AN, Poznyak AV, Sobenin IA et al. 2020. Mitochondrion as a selective target for the treatment of atherosclerosis: role of mitochondrial DNA mutations and defective mitophagy in the pathogenesis of atherosclerosis and chronic inflammation. CurrNeuropharmacol. 18(11):1064–1075. 10.2174/1570159X17666191118125018 PMC7709151 31744449

[cit0054] Parra V, Verdejo H, del Campo A et al. 2011. The complex interplay between mitochondrial dynamics and cardiac metabolism. J Bioenerg Biomembr. 43(1):47–51. 10.1007/s10863-011-9332-0 21258852 PMC3286637

[cit0055] Patel RP, Moellering D, Murphy-Ullrich J et al. 2000. Cell signaling by reactive nitrogen and oxygen species in atherosclerosis. Free Radical Biol Med. 28(12):1780–1794. 10.1016/s0891-5849(00)00235-5 10946220

[cit0056] Pathak RK, Kolishetti N, Dhar S. 2015. Targeted nanoparticles in mitochondrial Medicine. Wiley interdisciplinary reviews. Nanomedicine and nanobiotechnology. 7(3):315–329. 10.1002/wnan.1305 25348382 PMC4397104

[cit0057] Peng W, Cai G, Xia Y et al. 2019. Mitochondrial dysfunction in atherosclerosis. DNA Cell Biol. 38(7):597–606. 10.1089/dna.2018.4552 31095428

[cit0058] Pi X, Xie L, Patterson C. 2018. Emerging roles of vascular endothelium in metabolic homeostasis. Circ Res. 123(4):477–494. 10.1161/CIRCRESAHA.118.313237 30355249 PMC6205216

[cit0059] Protasoni M, Zeviani M. 2021. Mitochondrial structure and bioenergetics in normal and disease conditions. Int J Mol Sci. 22(2):586. 10.3390/ijms22020586 33435522 PMC7827222

[cit0060] Pulipaka S, Singuru G, Sahoo S et al. 2024. Therapeutic efficacies of mitochondria-targeted esculetin and metformin in the improvement of age-associated atherosclerosis via regulating AMPK activation. GeroScience. 46(2):2391–2408. 10.1007/s11357-023-01015-w 37968424 PMC10828355

[cit0061] Pulipaka S, Chempon H, Singuru G et al. 2024. Mitochondria-targeted esculetin and metformin delay endothelial senescence by promoting fatty acid beta-oxidation: relevance in age-associated atherosclerosis. Mech Ageing Dev. 219:111931. 10.1016/j.mad.2024.111931 38554949

[cit0062] Robichaux DJ, Harata M, Murphy E et al. 2023. Mitochondrial permeability transition pore-dependent necrosis. J Mol Cellular Cardiol. 174:47–55. 10.1016/j.yjmcc.2022.11.003 36410526 PMC9868081

[cit0063] Rodriguez-Cuenca S, Cocheme HM, Logan A et al. 2010. Consequences of long-term oral administration of the mitochondria-targeted antioxidant MitoQ to wild-type mice. Free Radical Biol Med. 48(1):161–172. 10.1016/j.freeradbiomed.2009.10.039 19854266

[cit0064] Rossman MJ, Santos-Parker JR, Steward CAC et al. 2018. Chronic supplementation with a mitochondrial antioxidant (MitoQ) improves vascular function in healthy older adults. Hypertension. 71(6):1056–1063. 10.1161/HYPERTENSIONAHA.117.10787 29661838 PMC5945293

[cit0065] Sacks B, Onal H, Martorana R et al. 2021. Mitochondrial targeted antioxidants, mitoquinone and SKQ1, not vitamin C, mitigate doxorubicin-induced damage in H9c2 myoblast: pretreatment vs. Co-treatment. BMC Pharmacol Toxicol. 22(1):49. 10.1186/s40360-021-00518-6 34530934 PMC8447656

[cit0066] Salnikova D, Orekhova V, Grechko A et al. 2021. Mitochondrial dysfunction in vascular wall cells and its role in atherosclerosis. Int J Mol Sci. 22(16):8990. 10.3390/ijms22168990 34445694 PMC8396504

[cit0067] Sato Y, Nakamura T, Yamada Y et al. 2024. The impact of, and expectations for, lipid nanoparticle technology: from cellular targeting to organelle targeting. J Control Release. 370:516–527. 10.1016/j.jconrel.2024.05.006 38718875

[cit0068] Scheffler IE. 2001. Mitochondria make a come back. Adv Drug Delivery Rev. 49(1-2):3–26. 10.1016/s0169-409x(01)00123-5 11377800

[cit0069] Shan Z, Wang Y, Qiu T et al. 2023. SS-31 alleviated nociceptive responses and restored mitochondrial function in a headache mouse model via Sirt3/Pgc-1alpha positive feedback loop. J Headache Pain. 24(1):65. 10.1186/s10194-023-01600-6 37271805 PMC10240765

[cit0070] Shemiakova T, Ivanova E, Grechko AV et al. 2020. Mitochondrial dysfunction and DNA damage in the context of pathogenesis of atherosclerosis. Biomedicines. 8(6):166. 10.3390/biomedicines8060166 32570831 PMC7344998

[cit0071] Singuru G, Pulipaka S, Shaikh A et al. 2024. Mitochondria targeted esculetin administration improves insulin resistance and hyperglycemia-induced atherosclerosis in db/db mice. J Mol Med (Berl). 102(7):927–945. 10.1007/s00109-024-02449-1 38758435

[cit0072] Stachowicz A, Wisniewska A, Czepiel K et al. 2024. Mitochondria-targeted hydrogen sulfide donor reduces atherogenesis by changing macrophage phenotypes and increasing UCP1 expression in vascular smooth muscle cells. Biomedicine & pharmacotherapy = Biomedecine & pharmacotherapie. 180:117527. 10.1016/j.biopha.2024.117527 39405912

[cit0073] Su X, Wang W, Ruan G et al. 2016. A comprehensive characterization of mitochondrial genome in papillary thyroid cancer. Int J Mol Sci. 17(10):1594. 10.3390/ijms17101594 27735863 PMC5085627

[cit0074] Szczesny B, Modis K, Yanagi K et al. 2014. AP39, a novel mitochondria-targeted hydrogen sulfide donor, stimulates cellular bioenergetics, exerts cytoprotective effects and protects against the loss of mitochondrial DNA integrity in oxidatively stressed endothelial cells in vitro. Nitric Oxide. 41:120–130. 10.1016/j.niox.2014.04.008 24755204 PMC4225488

[cit0075] Szeto HH. 2006. Cell-permeable, mitochondrial-targeted, peptide antioxidants. AAPS J. 8(2):E277–83. 10.1007/BF02854898 16796378 PMC3231562

[cit0076] Szewczyk A, Jarmuszkiewicz W, Koziel A et al. 2015. Mitochondrial mechanisms of endothelial dysfunction. Pharmacol Rep. 67(4):704–710. 10.1016/j.pharep.2015.04.009 26321271

[cit0077] Tanase DM Valasciuc E Gosav EM et al. 2023. Portrayal of NLRP3 inflammasome in atherosclerosis: current knowledge and therapeutic targets. Int J Mol Sci. 24(9):8162. 10.3390/ijms24098162 37175869 PMC10179095

[cit0078] Tasouli-Drakou V, Ogurek I, Shaikh T et al. 2025. Atherosclerosis: a comprehensive review of molecular factors and mechanisms. Int J Mol Sci. 26(3):1364. 10.3390/ijms26031364 39941130 PMC11818631

[cit0079] Tokuyama T, Yanagi S. 2023. Role of mitochondrial dynamics in heart diseases. Genes (Basel). 14(10):1876. 10.3390/genes14101876 37895224 PMC10606177

[cit0080] Tracy EP, Hughes W, Beare JE et al. 2021. Aging-induced impairment of vascular function: mitochondrial redox contributions and Physiological/Clinical implications. Antioxid Redox Signal. 35(12):974–1015. 10.1089/ars.2021.0031 34314229 PMC8905248

[cit0081] Tsujioka T, Sasaki D, Takeda A et al. 2021. Resveratrol-encapsulated mitochondria-targeting liposome enhances mitochondrial respiratory capacity in myocardial cells. Int J Mol Sci. 23(1):112. 10.3390/ijms23010112 35008537 PMC8745115

[cit0082] Vercellino I, Sazanov LA. 2022. The assembly, regulation and function of the mitochondrial respiratory chain. Nat Rev Mol Cell Biol. 23(2):141–161. 10.1038/s41580-021-00415-0 34621061

[cit0083] Wallace DC. 2018. Mitochondrial genetic Medicine. Nat Genet. 50(12):1642–1649. 10.1038/s41588-018-0264-z 30374071

[cit0084] Wang Y, Zang QS, Liu Z et al. 2011. Regulation of VEGF-induced endothelial cell migration by mitochondrial reactive oxygen species. Am J Physiol Cell Physiol. 301(3):C695–704. 10.1152/ajpcell.00322.2010 21653897 PMC3174570

[cit0085] Wang JY, Li JQ, Xiao YM et al. 2020. Triphenylphosphonium (TPP)-Based antioxidants: A new perspective on antioxidant design. ChemMedChem. 15(5):404–410. 10.1002/cmdc.201900695 32020724

[cit0086] Wang QH, Qin SW, Jiang JG. 2022. Improvement effects of esculetin on the formation and development of atherosclerosis. Biomedicine & pharmacotherapy = Biomedecine & pharmacotherapie. 150:113001. 10.1016/j.biopha.2022.113001 35658220

[cit0087] Wang Y, Fang D, Yang Q et al. 2023. Interactions between PCSK9 and NLRP3 inflammasome signaling in atherosclerosis. Front Immunol. 14:1126823. 10.3389/fimmu.2023.1126823 36911736 PMC9992811

[cit0088] Wang C, Yu B, Zhou H et al. 2025. tRF-AspGTC promotes intracranial aneurysm formation by controlling TRIM29-Mediated Galectin-3 ubiquitination. Research (Wash D C). 8:0574. 10.34133/research.0574 39776588 PMC11704088

[cit0089] Westermann B. 2002. Merging mitochondria matters: cellular role and molecular machinery of mitochondrial fusion. EMBO Rep. 3(6):527–531. 10.1093/embo-reports/kvf113 12052774 PMC1084147

[cit0090] Wongrakpanich A, Geary SM, Joiner ML et al. 2014. Mitochondria-targeting particles. Nanomedicine (Lond). 9(16):2531–2543. 10.2217/nnm.14.161 25490424 PMC4294695

[cit0091] Wu Y, Wang XH, Li XH et al. 2021. Common mtDNA variations at C5178a and A249d/T6392C/G10310A decrease the risk of severe COVID-19 in a han Chinese population from central China. Mil Med Res. 8(1):57. 10.1186/s40779-021-00351-2 34724985 PMC8558762

[cit0092] Wu C, Zhang Z, Zhang W et al. 2022. Mitochondrial dysfunction and mitochondrial therapies in heart failure. Pharmacol Res. 175:106038. 10.1016/j.phrs.2021.106038 34929300

[cit0093] Wu Y Jiang T Hua J et al. 2022. PINK1/Parkin-mediated mitophagy in cardiovascular disease: from pathogenesis to novel therapy. Int J Cardiol. 361:61–69. 10.1016/j.ijcard.2022.05.025 35594994

[cit0094] Xie S, Su E, Song X et al. 2023. GSDME in endothelial cells: inducing vascular inflammation and atherosclerosis via mitochondrial damage and STING pathway activation. Biomedicines. 11(9):2579. 10.3390/biomedicines11092579 37761020 PMC10526370

[cit0095] Xu H, She P, Zhao Z et al. 2023. Duplex responsive nanoplatform with cascade targeting for atherosclerosis photoacoustic diagnosis and multichannel combination therapy. Adv Mater. 35(21):e2300439. 10.1002/adma.202300439 36828777

[cit0096] Yamada Y, Akita H, Kamiya H et al. 2008. MITO-Porter: A liposome-based carrier system for delivery of macromolecules into mitochondria via membrane fusion. Biochim Biophys Acta. 1778(2):423–432. 10.1016/j.bbamem.2007.11.002 18054323

[cit0097] Yamada Y, Satrialdi, Hibino M et al. 2020. Power of mitochondrial drug delivery systems to produce innovative nanomedicines. Adv Drug Delivery Rev. 154-155:187–209. 10.1016/j.addr.2020.09.010 32987095

[cit0098] Yousif LF, Stewart KM, Horton KL et al. 2009. Mitochondria-penetrating peptides: sequence effects and model cargo transport. ChemBioChem. 10(12):2081–2088. 10.1002/cbic.200900017 19670199

[cit0099] Yu EP, Bennett MR. 2014. Mitochondrial DNA damage and atherosclerosis. Trends Endocrinol Metab. 25(9):481–487. 10.1016/j.tem.2014.06.008 25034130

[cit0100] Yu E, Calvert PA, Mercer JR et al. 2013. Mitochondrial DNA damage can promote atherosclerosis independently of reactive oxygen species through effects on smooth muscle cells and monocytes and correlates with higher-risk plaques in humans. Circulation. 128(7):702–712. 10.1161/CIRCULATIONAHA.113.002271 23841983

[cit0101] Yu T, Li X, Wang C et al. 2025. Lactylation of mitochondrial adenosine triphosphate synthase subunit alpha regulates vascular remodeling and progression of aortic dissection. Research (Wash D C). 8:0799. 10.34133/research.0799 40800583 PMC12342782

[cit0102] Zakirov FH, Zhang D, Grechko AV et al. 2020. Lipid-based gene delivery to macrophage mitochondria for atherosclerosis therapy. Pharmacology research & perspectives. 8(2):e00584. 10.1002/prp2.584 32237116 PMC7111069

[cit0103] Zhang M, Zhao H, Cai J et al. 2017. Chronic administration of mitochondrion-targeted peptide SS-31 prevents atherosclerotic development in ApoE knockout mice fed Western diet. PLoS One. 12(9):e0185688. 10.1371/journal.pone.0185688 28961281 PMC5621700

[cit0104] Zhang L Feng M Wang X et al. 2021. Peptide Szeto‑Schiller 31 ameliorates doxorubicin‑induced cardiotoxicity by inhibiting the activation of the p38 MAPK signaling pathway. Int J Mol Med. 47(4):63. 10.3892/ijmm.2021.4896 33649779 PMC7914074

[cit0105] Zhang Y, Weng J, Huan L et al. 2023. Mitophagy in atherosclerosis: from mechanism to therapy. Front Immunol. 14:1165507. 10.3389/fimmu.2023.1165507 37261351 PMC10228545

[cit0106] Zhang R, Jiang Y, Zhang G et al. 2025. Mitochondrial DNA in atherosclerosis: mechanisms, biomarker potential, and therapeutic perspectives. Int Immunopharmacol. 152:114449. 10.1016/j.intimp.2025.114449 40073813

[cit0107] Zhou F, Mei J, Yang S et al. 2020. Modified ZIF-8 nanoparticles attenuate osteoarthritis by reprogramming the metabolic pathway of synovial macrophages. ACS Appl Mater Interfaces. 12(2):2009–2022. 10.1021/acsami.9b16327 31849213

[cit0108] Zhou J, Li XY, Liu YJ et al. 2022. Full-coverage regulations of autophagy by ROS: from induction to maturation. Autophagy. 18(6):1240–1255. 10.1080/15548627.2021.1984656 34662529 PMC9225210

[cit0109] Zhou H, Wang C, Wang W. 2025. CircGNAQ promotes intracranial aneurysm formation by facilitating vascular smooth muscle cell phenotypic switching and apoptosis. Antioxid Redox Signal. Epub ahead of print. 10.1177/15230864251380271 40986450

[cit0110] Zielonka J, Joseph J, Sikora A et al. 2017. Mitochondria-targeted triphenylphosphonium-based compounds: syntheses, mechanisms of action, and therapeutic and diagnostic applications. Chem Rev. 117(15):10043–10120. 10.1021/acs.chemrev.7b00042 28654243 PMC5611849

[cit0111] Zinovkin RA, Zamyatnin AA. 2019. Mitochondria-targeted drugs. Curr Mol Pharmacol. 12(3):202–214. 10.2174/1874467212666181127151059 30479224 PMC6875871

[cit0112] Zmijewski JW, Moellering DR, Goffe CLe et al. 2005. Oxidized LDL induces mitochondrially associated reactive oxygen/nitrogen species formation in endothelial cells. Am J Physiol Heart Circ Physiol. 289(2):H852–61. 10.1152/ajpheart.00015.2005 15805232

[cit0113] Zorko M, Langel U. 2022. Cell-penetrating peptides. Methods Mol Biol. 2383:3–32. 10.1007/978-1-0716-1752-6_1 34766279

[cit0114] Zorov DB, Filburn CR, Klotz LO et al. 2000. Reactive oxygen species (ROS)-induced ROS release: a new phenomenon accompanying induction of the mitochondrial permeability transition in cardiac myocytes. J Exp Med. 192(7):1001–1014. 10.1084/jem.192.7.1001 11015441 PMC2193314

[cit0115] Zorov DB, Juhaszova M, Sollott SJ. 2006. Mitochondrial ROS-induced ROS release: an update and review. Biochim Biophys Acta. 1757(5-6):509–517. 10.1016/j.bbabio.2006.04.029 16829228

[cit0116] Zorov DB, Juhaszova M, Sollott SJ. 2014. Mitochondrial reactive oxygen species (ROS) and ROS-induced ROS release. Physiol Rev. 94(3):909–950. 10.1152/physrev.00026.2013 24987008 PMC4101632

